# Flip/flop mating-type switching in the methylotrophic yeast *Ogataea polymorpha* is regulated by an Efg1-Rme1-Ste12 pathway

**DOI:** 10.1371/journal.pgen.1007092

**Published:** 2017-11-27

**Authors:** Sara J. Hanson, Kevin P. Byrne, Kenneth H. Wolfe

**Affiliations:** 1 UCD Conway Institute, School of Medicine, University College Dublin, Dublin 4, Ireland; 2 Department of Molecular Biology, Colorado College, Colorado Springs, Colorado, United States of America; Duke University Medical Center, UNITED STATES

## Abstract

In haploid cells of *Ogataea (Hansenula) polymorpha* an environmental signal, nitrogen starvation, induces a reversible change in the structure of a chromosome. This process, mating-type switching, inverts a 19-kb DNA region to place either *MAT***a** or *MAT*α genes under centromeric repression of transcription, depending on the orientation of the region. Here, we investigated the genetic pathway that controls switching. We characterized the transcriptomes of haploid and diploid *O*. *polymorpha* by RNAseq in rich and nitrogen-deficient media, and found that there are no constitutively **a**-specific or α-specific genes other than the *MAT* genes themselves. We mapped a switching defect in a sibling species (*O*. *parapolymorpha* strain DL-1) by interspecies bulk segregant analysis to a frameshift in the transcription factor *EFG1*, which in *Candida albicans* regulates filamentous growth and white-opaque switching. Gene knockout, overexpression and ChIPseq experiments show that *EFG1* regulates *RME1*, which in turn regulates *STE12*, to achieve mating-type switching. All three genes are necessary both for switching and for mating. Overexpression of *RME1* or *STE12* is sufficient to induce switching without a nitrogen depletion signal. The homologous recombination genes *RAD51* and *RAD17* are also necessary for switching. The pathway controlling switching in *O*. *polymorpha* shares no components with the regulation of *HO* in *S*. *cerevisiae*, which does not involve any environmental signal, but it shares some components with mating-type switching in *Kluyveromyces lactis* and with white-opaque phenotypic switching in *C*. *albicans*.

## Author summary

The molecular mechanisms of self-fertility (homothallism) vary enormously among fungal species. We previously found that in the yeast *Ogataea polymorpha*, homothallism is achieved by a novel mating-type switching mechanism that exchanges the locations of *MAT***a** and *MAT*α genes between expression and repression contexts. Switching in this species is induced by nitrogen depletion, unlike the analogous process in *Saccharomyces cerevisiae*. Here, we show that the upstream parts of the genetic pathway controlling the environmental induction of switching in *O*. *polymorpha* are the same as the environmental pathway that induces competence for mating in this species.

## Introduction

In yeast species (unicellular fungi) that can reproduce sexually, the ability of a cell to mate with other cells is governed by which mating-type genes it expresses [1, 2]. In ascomycete yeasts, these genes are located at a single genomic site called the mating-type (*MAT*) locus. Mating generally occurs between two haploid cells with opposite genotypes (*MAT***a** and *MAT*α) at this locus, to form a diploid zygote (*MAT***a**/α). In some ascomycete yeasts such as *Saccharomyces cerevisiae*, haploid cells are able to change their *MAT* genotypes by a process called mating-type switching [3, 4]. During this process, DNA at the *MAT* locus is physically replaced, exchanging a *MAT***a** allele for a *MAT*α allele or vice versa. Mating-type switching is a form of secondary homothallism [5] because it enables a yeast strain to mate with any other strain of the same species, regardless of their initial mating types, by means of fusion between **a**-cells and α-cells [6, 7].

**Fig 1:**
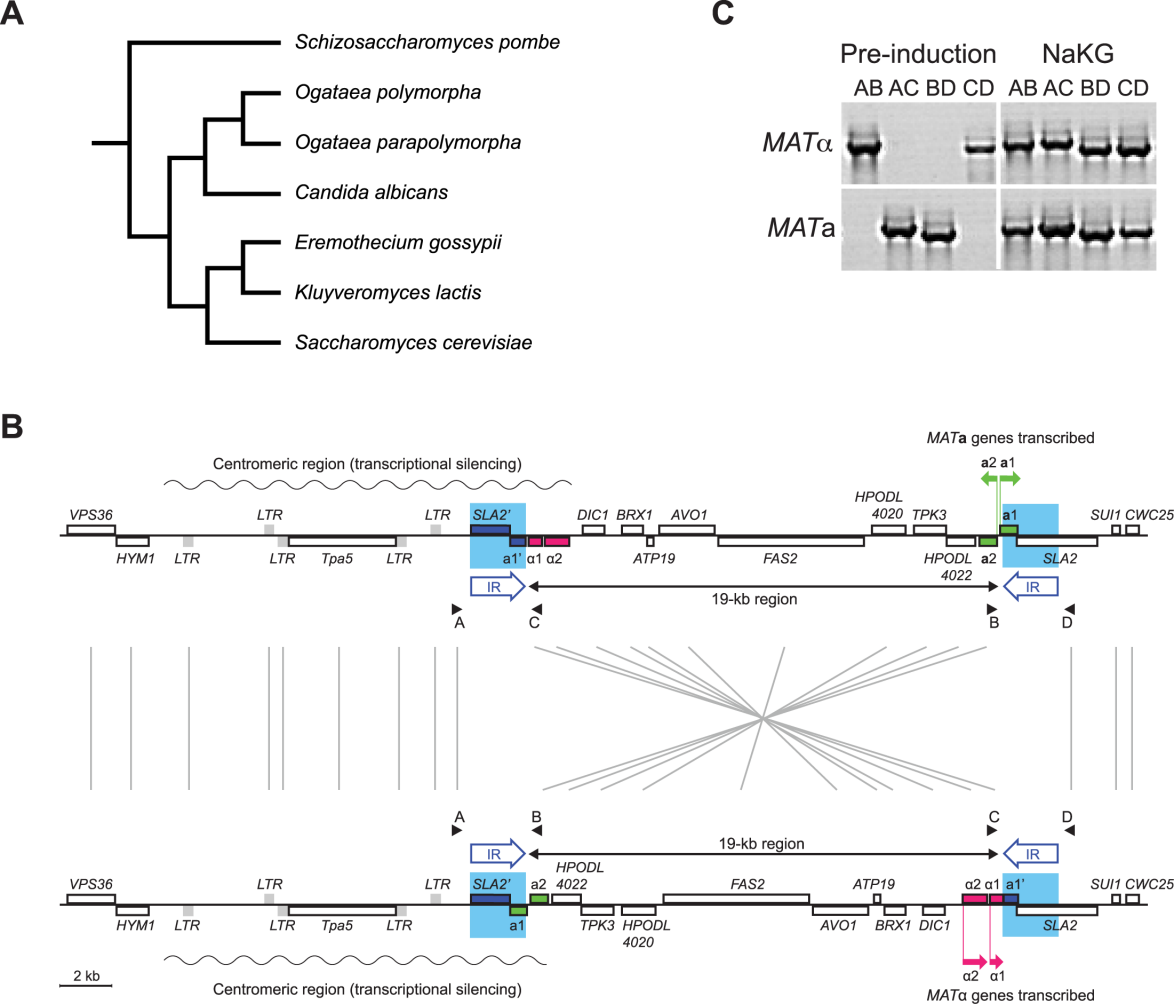
Mating-type switching in *O*. *polymorpha* occurs through an inducible flip/flop chromosomal inversion mechanism [10, 11]. **(A)** Phylogenetic relationship among the yeast species compared in this study. The tree is re-drawn from the phylogenomic study by Shen *et al*. [67] and is a cladogram, i.e. branch lengths are not proportional to divergence times. All the species are in subphylum Saccharomycotina except for *Schizosaccharomyces pombe*, which is in Taphrinomycotina. **(B)** Structure of the *MAT* region on chromosome 3 of *O*. *polymorpha* NCYC495 [12] in its two possible orientations. In each orientation, only the *MAT* genes far from the centromere are expressed. The upper part shows the orientation in which *MAT***a** genes are transcribed (green arrows), and the lower part shows the orientation in which *MAT*α genes are transcribed (magenta arrows). Wavy lines indicate the approximate extent of transcriptional silencing around the centromere. Cyan rectangles and the arrows labeled IR represent the two identical copies of a 2-kb sequence that form the Inverted Repeat. Small rectangles represent genes: intact *MAT***a** genes (green), intact *MAT*α genes (magenta), other genes (white), or LTRs of the Ty5-like retrotransposon Tpa5 (gray). The dark blue regions labeled *SLA2*’ and **a**1’ are non-functional truncated copies of parts of the *SLA2* and **a**1 genes, located in the IR. Genes drawn above or below the horizontal lines are transcribed rightwards or leftwards, respectively. Arrowheads A-D indicate the locations of PCR primers used to determine mating-type. **(C)** Induction of mating-type switching by nitrogen depletion. *MAT*α strains (upper) and *MAT***a** strains (lower) were grown in YPD ‘pre-induction’ cultures, and then transferred into NaKG media, which contains no nitrogen source and strongly reduces growth rate. DNA samples were taken from the pre-induction cultures and 24 h after transfer into NaKG. PCR amplifications were performed with the primer combinations indicated. The PCR product sizes are 2.6 kb (AB), 2.7 kb (AC), 2.3 kb (BD) and 2.3 kb (CD).

The molecular mechanism and regulation of mating-type switching in *S*. *cerevisiae* has been elucidated by extensive studies over the past several decades and is well understood [3, 8]. It involves an endonuclease (HO) that cuts the outgoing *MAT* locus, and two ‘silent cassettes’ (*HMR* and *HML*) that contain unexpressed copies of the *MAT***a** and *MAT*α DNA sequences. One of the cassettes is chosen to be used as the template for synthesis of new DNA to repair the *MAT* locus, replacing *MAT* with a sequence of the opposite genotype. In contrast, until recently little was known about how other ascomycete yeasts switch mating types, other than in *Schizosaccharomyces pombe* [9] which is a member of a different subphylum. In 2014, Maekawa and Kaneko [10], and our group [11], discovered that haploid cells of *Ogataea polymorpha* switch mating types by a novel ‘flip/flop’ mechanism that is quite different from the mechanism used by *S*. *cerevisiae*. *O*. *polymorpha* (formerly called *Hansenula polymorpha*) is a methylotrophic yeast in the same subphylum as *S*. *cerevisiae* (Saccharomycotina, the budding yeasts) but quite distantly related to it (Fig 1A).

*O*. *polymorpha* chromosome 3 contains both a *MAT***a** locus and a *MAT*α locus, approximately 19 kb apart (Fig 1B). The two *MAT* loci are beside two copies of an identical 2-kb DNA sequence that form an inverted repeat (IR) on the chromosome. During mating type switching, the two copies of the IR recombine, inverting the orientation of the 19-kb region relative to the rest of the chromosome. The centromere of chromosome 3 is located just to the left of the left copy of the IR (Fig 1B). The *MAT* locus proximal to the centromere is not transcribed, probably due to silencing by centromeric heterochromatin, whereas the distal *MAT* locus is transcribed. By inverting the 19-kb region, mating type switching swaps the locations of the *MAT***a** and *MAT*α genes, repressing the *MAT* genes that were previously expressed, and expressing the ones that were previously repressed. Similar flip/flop mating type switching mechanisms are now known in three other Saccharomycotina species (*Komagataella phaffii*, *Pachysolen tannophilus*, and *Ascoidea rubescens*) [4, 11, 12].

Mating type switching in *O*. *polymorpha* is induced by an environmental signal, nitrogen depletion [10, 11]. In a culture transferred into media that contains no nitrogen, up to approximately 25% the cells in the culture switch their mating type (Fig 1C). This situation, in which an environmental signal reproducibly induces a DNA rearrangement at a specific chromosomal locus, is unusual in biology and we were motivated to investigate its mechanism. Our aim in the current study was to identify the pathway in *O*. *polymorpha* that detects the environmental signal and executes rearrangement of chromosome 3 in response. *A priori*, we know that the pathway in *O*. *polymorpha* must be quite different from the pathway that regulates mating-type switching in *S*. *cerevisiae* [3, 13], because switching in *S*. *cerevisiae* is not regulated by the environment and occurs even in rich media, and because *O*. *polymorpha* has no ortholog of the *S*. *cerevisiae* HO endonuclease gene. Therefore, both the upstream (nitrogen-sensing) and downstream (DNA inversion) parts of the pathway in *O*. *polymorpha* must be different from *S*. *cerevisiae*. Furthermore, since the DNA rearrangements that occur during switching in *S*. *cerevisiae*, *O*. *polymorpha* and *Kluyveromyces lactis* are all substantially different but are descendants of a common ancestral switching mechanism [4, 14, 15], we were interested to determine how the pathways that regulate these rearrangements have evolved.

To identify components of the switching pathway in *O*. *polymorpha*, we used several strategies including transcriptomic analysis, candidate gene approaches, and mapping the defective gene in a naturally-occurring mutant that is unable to switch mating types. We identified five genes that are required for switching. Although we were unable to deduce all the steps that lead from nitrogen depletion to mating type switching, we infer that *O*. *polymorpha* senses nitrogen depletion using the Protein Kinase A (PKA) pathway, which then transmits a signal via Ste12 to induce mating and/or mating type switching, and that recombination between the IRs is mediated by the homologous recombination pathway for DNA repair. We compare the roles of genes in the *O*. *polymorpha* pathway to the roles of their orthologs in other species.

## Results

### Transcriptomic response of *O*. *polymorpha* to nitrogen depletion

Our initial approach to search for genes involved in mating-type switching in *O*. *polymorpha* was to look for differences between the transcriptomes of cells that are switching and cells that are not switching. Switching in several methylotrophic yeast species is induced by nitrogen depletion [10–12, 16], and in *O*. *polymorpha* we used liquid NaKG media (0.5% NaOAc, 1% KCl, 1% glucose), which completely lacks amino acids or any other source of nitrogen, to induce switching. *O*. *polymorpha* grows poorly in NaKG, so to induce switching we first grew ‘pre-induction’ cultures in rich media (YPD) and then transferred the cells, after washing, into NaKG. In the haploid strain NCYC495, recombination between the IRs in the *MAT* region was induced within 24 hours after transfer into NaKG, whereas no recombination occurred in the YPD pre-induction cultures (Fig 1C).

To examine the transcriptional response induced by nitrogen depletion, we used mRNAseq to compare the transcriptomes of *O*. *polymorpha* cells 2 h after transfer from a YPD pre-induction culture into NaKG, to parallel cultures transferred into fresh YPD. Furthermore, because we expect that switching occurs only in haploid cells, we conducted this experiment in parallel on haploid (*MAT***a** and *MAT*α isogenic strains) and diploid (*MAT***a**/α) cells.

Growth of all three cell types in NaKG resulted in a robust transcriptional response to nitrogen depletion, with a large number of genes significantly up- or down-regulated relative to YPD (S1 Fig; S1 Table). Regardless of cell type, homologs of *S*. *cerevisiae* genes for nitrogen starvation responses were induced, such as transporters of amino acids (*DIP5*, *GAP1*), urea (*DUR3*), and allantoate (*SEO1*), and amidases for the release of amide groups from urea (*DUR1*,*2*), pyrimidines (*PYD3*), or other substrates (*AMD2*). Ribosomal protein genes were strongly repressed, as expected because of the reduced growth rate in NaKG (S1 Table). However, orthologs of *S*. *cerevisiae* genes with mating or sporulation functions were not induced by these nitrogen depletion conditions alone, even though mating (of haploids) and sporulation (of diploids) can be induced by plating cells onto similar nitrogen-depleted solid media [17]. Among the genes strongly upregulated in NaKG were two transcription factors, *RME1* and *CZF1-like3* (one of three *O*. *polymorpha* co-orthologs of *C*. *albicans CZF1*, which is a singleton zinc finger gene with no *S*. *cerevisiae* ortholog [18]). Both of these genes were uniformly induced in all three cell types (*MAT***a**, *MAT*α and *MAT***a**/α), with *CZF1-like3* upregulated 69- to 93-fold, and *RME1* upregulated 26- to 79-fold, upon transfer into NaKG (S1 Table).

### Lack of constitutive *MAT*a- and *MAT*α-specific genes

In *S*. *cerevisiae*, defined sets of **a**- and α-specific genes that allow haploid cells to identify and respond to the presence of a mating partner are well established [19]. These genes are constitutively expressed in *S*. *cerevisiae* cells of the appropriate mating type. Surprisingly, comparison of gene expression between haploid *O*. *polymorpha* **a**-cells and α-cells in either NaKG or YPD media revealed that there are essentially no constitutive **a**- or α-specific genes in this species, apart from the *MAT* genes themselves (S1 Fig; S2 Fig). All haploid cells of *O*. *polymorpha* contain four *MAT* genes (*MAT* α1, α2, **a**1, and **a**2), and the orientation of the 19-kb region specifies whether the α1 and α2 genes, or the **a**1 and **a**2 genes, are placed at the expression site (Fig 1B). In NaKG, transcription of α1 and α2 was respectively 53-fold and 39-fold higher in α-cells than in **a**-cells; **a**2 was 31-fold lower, and **a**1 was just 2-fold lower. In YPD, α1 and α2 were 4-fold and 9-fold higher, **a**1 was 6-fold lower, and **a**1 showed no difference. *KAR4*, which in *S*. *cerevisiae* is a general pheromone-induced gene [20] required for fusion of the haploid nuclei after mating, showed moderately higher expression in **a**-cells than in α-cells (2 to 3-fold; S2 Fig). No other genes showed more than a 2-fold difference in transcription between **a**- and α-cells, in either of the two media (S2 Fig; S2 Table). This result contrasts sharply with *S*. *cerevisiae*, where for example several **a**-specific genes such as *MFA2*, *STE2* and *BAR1* have more than 10-fold higher expression in *MAT***a** than *MAT*α cells in YPD [19]. It is also consistent with previous observations that expression of the pheromone receptors *STE2* and *STE3* in haploid *O*. *polymorpha* is independent of cell type [10].

These experiments also enabled us to identify gene expression differences between haploid and diploid cells. *O*. *polymorpha* is haplontic, and its diploid state is normally transient because meiosis is induced by the same conditions (nitrogen depletion) that induce mating. However, diploids can be maintained stably on YPD. We calculated the haploid-to-diploid expression ratio for each gene as the ratio between its transcription in **a**-cells and **a**/α-cells. The values of this ratio in different genes were quite consistent between YPD and NaKG media (Pearson’s *R* = 0.68; S3 Fig). Among the genes showing the strongest bias in YPD towards haploid-specific expression were several transcription factors including *CZF1-like1*, *CZF1-like2*, *CRZ1*, *GAT1*, and *MGA1* (S3 Table). Of these, only *CZF1-like2* was also haploid-specific in NaKG. Transcription factor *DAL81* appeared diploid-specific in both media (S3 Table).

Because mating-type switching occurs in haploid cells grown in NaKG, but not in haploids grown in YPD, and presumably not in diploids, we anticipated that genes with roles in switching might be identifiable as transcripts that are both haploid-specific and NaKG-specific. However, analysis of the genes fitting this transcription profile did not reveal any strong candidates for the downstream steps in the switching process, such as DNA recombination or endonuclease genes. Instead, most of the genes with this profile had metabolic functions (S4 Fig). The most haploid-specific and diploid-specific genes in the two media are listed in S3 Table.

### A switching defect in *O*. *parapolymorpha* strain DL-1 maps to *EFG1*

**Fig 2:**
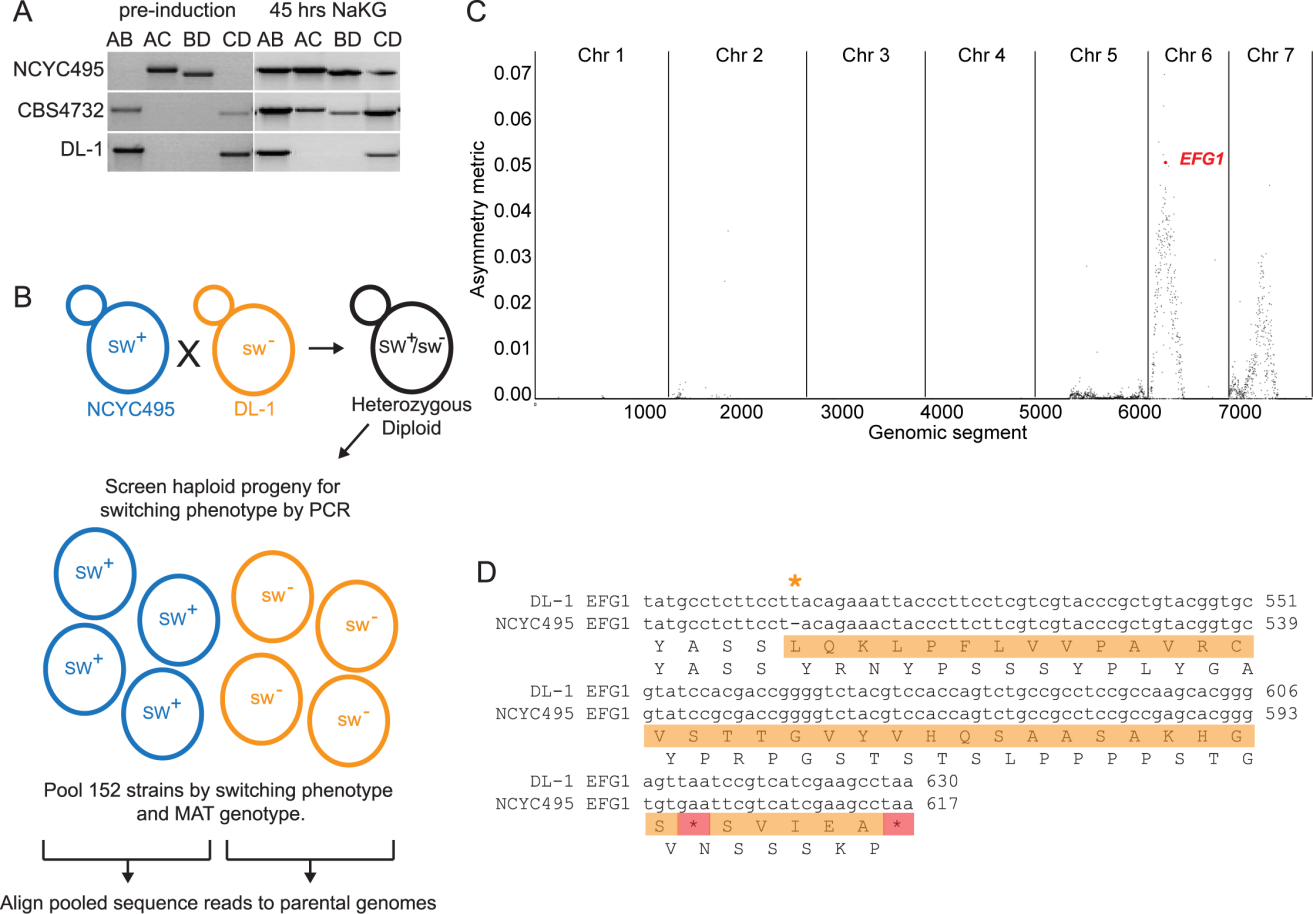
A switching defect in *O*. *parapolymorpha* strain DL-1 maps to the transcription factor *EFG1*. **(A)** PCR determination of *MAT* genotypes in *O*. *parapolymorpha* strain DL-1 and *O*. *polymorpha* strains NCYC495 and CBS4732, before and after 45 h growth in NaKG media. PCR primers A-D are as in Fig 1. **(B)** Experimental design for bulk segregant analysis of the DL-1 switching defect. Switching competent (blue) and non-switching (orange) backgrounds were crossed to form a heterozygous diploid. The diploid was sporulated and individual haploid progeny were tested for the ability to switch after growth for 24 h in NaKG. The haploids were pooled by their switching phenotypes prior to genome sequencing. **(C)** Regions of the genome differentially inherited by switching and non-switching progeny. The peak on chromosome 6 contains the *EFG1* locus, indicated by the red dot. Genomic segments correspond to single genes or intergenic regions. **(D)** The *EFG1* locus in *O*. *parapolymorpha* strain DL-1 contains a 1-bp insertion. The nucleotide sequence alignment shows the extra T (orange asterisk) in DL-1, causing a frameshift (orange) leading to premature stop codons (red) and a truncated protein product relative to *O*. *polymorpha* strain NCYC495.

Next, in an alternative approach to find a component of the switching pathway, we made use of a naturally occurring mutant. When assaying the *MAT* genotypes of *Ogataea* strains, we discovered that strain DL-1 is unable to switch mating-types, even after 45 h growth in NaKG, in contrast to strains NCYC495 and CBS4732 (Fig 2A). Strain DL-1 has previously been described as ‘semi-sterile,’ meaning that it is very inefficient at forming diploids under nutrient-limited conditions [21]. The semi-sterility phenotype of DL-1 is therefore likely due to a loss of the signal that is induced by nitrogen depletion, upstream of the steps that normally lead to either mating or switching in response to the signal.

Strains DL-1, NCYC495 and CBS4732 were all historically classified as *Hansenula polymorpha* but it has recently been recognized, based on sequence divergence, that DL-1 is a different species from the other two. DL-1 is now classified as *Ogataea parapolymorpha*, whereas NCYC495 and CBS4732 are *O*. *polymorpha* [22, 23]. However, the genome sequence of *O*. *parapolymorpha* DL-1 [24] is completely collinear with the genome sequence of *O*. *polymorpha* NCYC495 [12]. Both species have 7 chromosomes, and there are no translocations or other chromosomal rearrangements between them, even though the genomes are approximately 10% different in nucleotide sequence [11]. The fact that the genomes are collinear suggested to us that the mating-type switching defect in DL-1 could be mapped by using an interspecies genetic cross between it and the *O*. *polymorpha* laboratory strain NCYC495.

We used bulk segregant analysis [25] to map the locus causing the switching defect. We first isolated a rare diploid from a cross between DL-1 (*leu2 ura3* genotype) and an NCYC495 derivative (*ade11 met6* genotype), selecting for prototrophy. We then sporulated the diploid and isolated haploid segregants grown from random spores (Fig 2B). Segregants were screened individually for their ability to switch mating-types after 24 h in NaKG by the same PCR assay used above. We made four pools of segregants: *MAT***a** switchers, *MAT***a** non-switchers, *MAT*α switchers, and *MAT*α non-switchers, each pool containing between 30 and 52 haploid clones (S5A Fig), and sequenced each pool. Cultures of each clone in a pool were grown individually and then combined into pools in equal cell numbers for DNA extraction and genome sequencing. The sequence reads from the switching and non-switching pools were then mapped to the parental DL-1 and NCYC495 genome sequences (S5B Fig); only reads that were unambiguously derived from one identifiable parent were mapped. We developed an asymmetry metric (see Methods) to detect regions of the genome where biased inheritance of parental alleles correlated with the switching/non-switching phenotype in the expected direction (Fig 2C).

Two peaks of asymmetrical inheritance were detected (Fig 2C). The strongest signal was located on chromosome 6 and was centered near the gene *OPOL_95241*, which we refer to as *O*. *polymorpha EFG1*. It is orthologous to the *C*. *albicans* transcription factor *EFG1* [26] and to the *S*. *cerevisiae* gene pair *PHD1* and *SOK2* derived from the Whole-Genome Duplication [27–29]. Comparison of the *EFG1* sequences from the parental NCYC495 and DL-1 genomes revealed a single-base insertion at nucleotide 512 in the DL-1 gene that causes a frameshift (Fig 2D). The predicted DL-1 Efg1 protein product is truncated to 203 residues, compared to 437 residues in NCYC495. The DL-1 Efg1 protein lacks a DNA-binding domain (APSES domain [26, 30, 31]) that is conserved among Efg1 orthologs in multiple species including *C*. *albicans* and *S*. *cerevisiae* (S6A Fig). A second region of asymmetrical inheritance occurred on chromosome 7 near coordinate 330 kb (Fig 2C). Comparison of the NCYC495 and DL-1 genomes in this region did not reveal any candidate disabling mutations in genes, or differences in gene content. Considering that this analysis used a cross between two different species, it is possible that the chromosome 7 region contains a gene that interacts with a gene near *EFG1*, for which an interspecies combination of alleles is inviable, but we did not investigate this region further.

**Fig 3:**
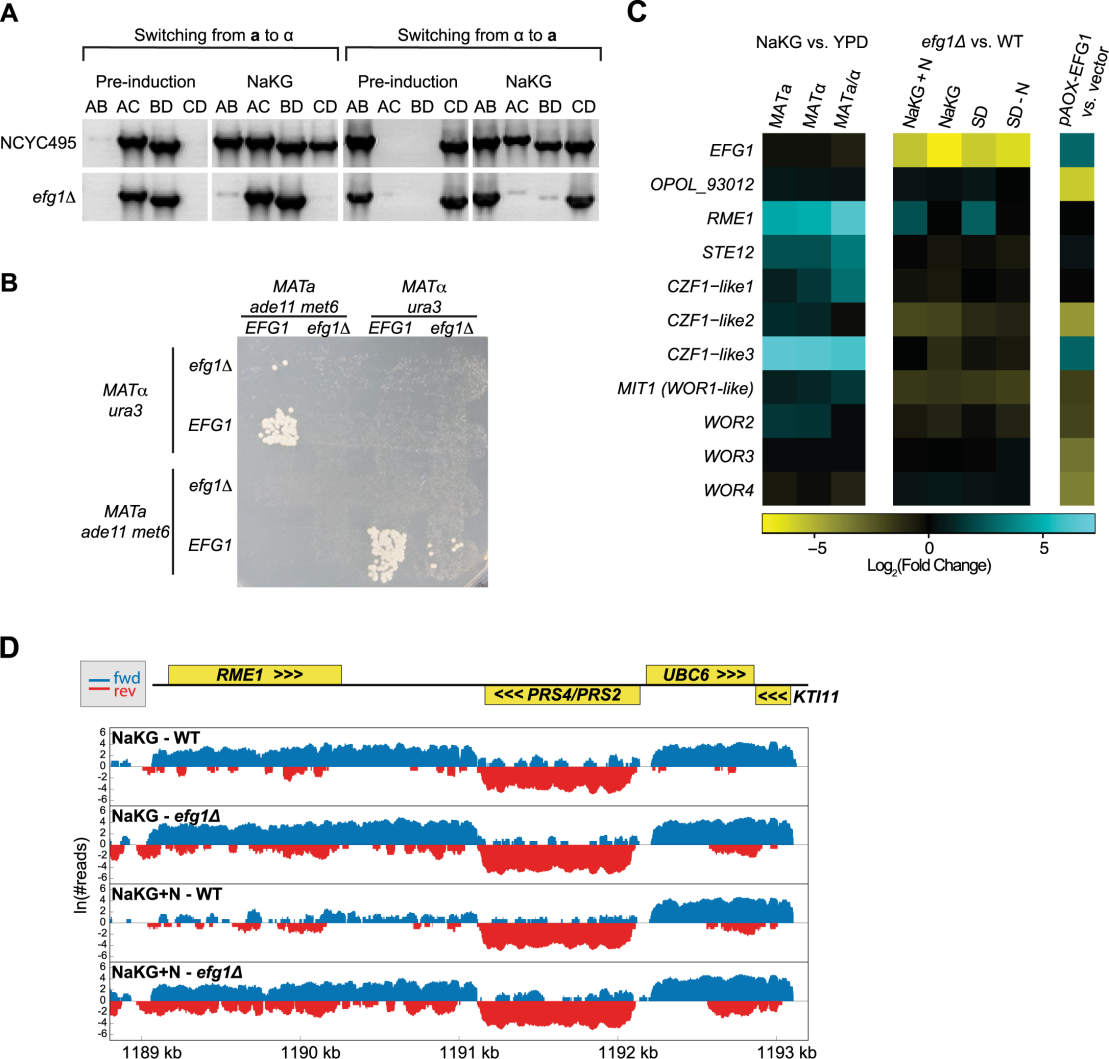
*EFG1* is required for *O*. *polymorpha* mating-type switching and mating. **(A)** PCR determination of *MAT* genotypes before and after 24 h growth in NaKG of *efg1*Δ and wild-type NCYC495 strains. *efg1*Δ strains were constructed in both *MAT***a** and *MAT*α backgrounds. PCR primers A-D are as in Fig 1. **(B)** Photographs of diploid growth on SD plates following crosses of haploids on MEMA for 24 h. **(C)** Regulation of transcription factor expression by *EFG1* and by cell type. The heatmaps show log_2_(Fold Change) in expression of the genes named on the left, measured by mRNAseq, for the pairs of conditions named on the top. Columns 1–3 compare gene expression in nitrogen-poor (NaKG) to nitrogen-rich (YPD) media, in haploid *MAT***a**, haploid *MAT*α, and diploid *MAT***a**/α strains. Columns 4–7 compare *efg1Δ* to wildtype strains, in two nitrogen-rich (NaKG+N, SD) and two nitrogen-poor (NaKG, SD-N) media. Column 8 compares a *pAOX-EFG1* overexpression strain to a strain containing an empty *pAOX* vector, after methanol induction of *pAOX*. **(D)** *EFG1* represses *RME1* in rich media. The genomic region containing *RME1* on chromosome 3 is shown. Numbers of mapped RNAseq reads are plotted (log scale) for two strains (wildtype and *efg1Δ*) in two conditions (nitrogen-poor NaKG, and nitrogen-rich NaKG+N). Blue and red indicate transcription in the forward and reverse directions, respectively. Yellow boxes show the coding regions of genes. *RME1* has a long 3’ untranslated region of approximately 850 bp.

To confirm that *EFG1* plays a role in mating-type switching in *O*. *polymorpha*, we deleted it from both *MAT***a** and *MAT*α strains. Gene deletions were made in *ku80Δ* derivatives from the NCYC495 genetic background [32]. PCR assays showed that, after 24 h in NaKG, almost no switched *MAT* locus products were formed in the *efg1Δ* strains, whereas extensive switching occurred in the wildtype control strains (Fig 3A). Furthermore, the *efg1Δ* strains were defective in mating, similar to the semi-sterility phenotype of DL-1. Crosses of *efg1Δ* x *efg1Δ* strains yielded no progeny, and crosses of *efg1Δ* x *EFG1* strains yielded only a small number of progeny compared to wildtype crosses (Fig 3B). This result indicates that both parents in a cross require *EFG1* activity in order to mate.

### An *EFG1-*dependent mechanism represses *RME1* in nitrogen-rich conditions

Because *EFG1* is required for both mating and mating-type switching in *O*. *polymorpha* (Fig 3A and 3B), we reasoned that it must act in an upstream part of the nutrient-sensing pathway that is shared by these two processes. Such a function is consistent with the known role of *EFG1* in *C*. *albicans* as the major transcription factor of the PKA pathway [33–36], even though its *S*. *cerevisiae* orthologs *PHD1* and *SOK2* have no role in mating or switching. We therefore searched for *O*. *polymorpha* genes whose expression depends on Efg1.

To find genes downstream of *EFG1*, we compared the transcriptional profiles of wildtype and *efg1Δ* haploid strains, by mRNAseq in nitrogen-poor and nitrogen-rich conditions. We used two different types of paired media for this experiment. One was a comparison of transcriptomes in NaKG versus NaKG plus 40 mM ammonium sulfate, which we have previously shown abolishes switching [11]. The other, chosen to try to reduce the strong general nutrient depletion signal we observed with NaKG (S1 Fig), was a comparison of transcriptomes in synthetic defined media (SD, which includes 40 mM ammonium sulfate) versus SD lacking this nitrogen source. These mRNAseq experiments identified many nutrient transporters and enzymes that have *EFG1-*dependent expression in nitrogen-poor media (S4 Table), consistent with *EFG1*’s expected role in the PKA pathway, but no obvious candidates for direct actors in the flip/flop inversion mechanism, such as DNA recombinases or endonucleases. However, these experiments also showed that one of the genes with the largest *EFG1-*dependent differences in expression between nitrogen-rich and nitrogen-poor conditions was another transcription factor, *RME1*.

*O*. *polymorpha RME1* is a gene that is substantially more highly transcribed in nitrogen-poor than in nitrogen-rich conditions, being among the top 2% of genes upregulated in NaKG (Fig 3C; S1 Table). In nitrogen-rich conditions, one of the strongest effects of deleting *EFG1* was to increase the expression of *RME1* from its low baseline, by factors of 4.4-fold in NaKG + ammonium sulfate, and 6.6-fold in SD, relative to wildtype cells (Fig 3C and 3D). In contrast, in nitrogen-poor conditions *RME1* expression was high and unchanged between *efg1Δ* and wildtype cells (Fig 3D; S4 Table). In the *efg1Δ* strain, *RME1* was expressed in both nitrogen-rich and poor media. Thus, transcription of *RME1* in nitrogen-rich conditions is normally repressed by an *EFG1*-dependent mechanism, which could either be direct or involve intermediate proteins. *EFG1* itself showed no difference in expression between NaKG and YPD (Fig 3C). In *Kluyveromyces lactis*, *RME1* (also called *MTS1*) is required for both mating-type switching and mating [14, 37], so regulation of *RME1* by *EFG1* in *O*. *polymorpha* therefore suggests a mechanism connecting the nitrogen limitation response to switching and mating. In contrast, the main function of *RME1* in *S*. *cerevisiae* is as a repressor of meiosis via repression of *IME1*, which has no ortholog in *O*. *polymorpha* [38, 39].

The set of genes showing expression changes in the *efg1Δ* strain also included some ‘white-opaque circuit’ genes. These genes are *O*. *polymorpha* homologs of genes that form a feed-forward circuit in *C*. *albicans* governing the phenotypic switch between mating-competent (opaque) and mating-incompetent (white) cell states. The *C*. *albicans* circuit includes *EFG1*, *WOR1*, *WOR2* and *CZF1* [40]. *O*. *polymorpha* has no ortholog of *C*. *albicans WOR1*, but has a paralogous gene (*OPOL_7784*) that we refer to as *MIT1* because of its similarity to *S*. *cerevisiae MIT1* [41]. Among the strongest effects of deleting *O*. *polymorpha EFG1* were decreases of expression of *CZF1-like2* and *MIT1*, which occurred in both nitrogen-poor and nitrogen-rich conditions (Fig 3C; S4 Table; all decreases were by less than 4-fold).

### *EFG1* overexpression represses an *EFG1* paralog

In parallel to the experiments with the *efg1Δ* deletion strain, we also used an overexpression strain to search for genes regulated by *EFG1*. We placed *EFG1* under the control of the *O*. *polymorpha* alcohol oxidase promoter (*pAOX*). Expression from *pAOX* is robustly induced when cells are switched from growth in glucose to growth in media containing methanol as the carbon source. We used mRNAseq to compare the transcriptome of the *pAOX-EFG1* strain to a control strain containing an empty *pAOX* construct, after overnight growth in methanol media. Methanol induced 7-fold higher transcription of *EFG1* in the *pAOX-EFG1* strain than in the control (Fig 3C). Overexpression of *EFG1* led to changes in transcription of large numbers of genes (221 genes downregulated, and 26 genes upregulated, by factors of at least 8-fold; S5A Table). Notably, *RME1* transcription remained unchanged in the *EFG1* overexpression strain (Fig 3C).

One of the strongest effects of overexpressing *EFG1* (*OPOL_95241*) was 53-fold repression of a related gene, *OPOL_93012* (Fig 3C). Phylogenetic analysis showed that these two APSES domain proteins are the products of a gene duplication that occurred within the genus *Ogataea* (S6B Fig). This gene duplication is separate from an older duplication that formed the *EFG1* homolog *EFH1* in the *Candida* clade [42]. The *O*. *polymorpha* Efg1 and OPOL_93012 proteins have 49% amino acid sequence identity. The sister species *O*. *parapolymorpha* has orthologs of both *EFG1* (with a frameshift) and *OPOL_93012*, but other budding yeasts including methylotrophs outside the genus *Ogataea* have only a single gene. *EFG1* and *OPOL_93012* are both transcribed in both nitrogen-poor and nitrogen-rich conditions. *EFG1* has higher expression in haploids than in diploids (2.8- to 3.7-fold), whereas *OPOL_93012* shows little difference between cell types (S3 Table). Expression of *OPOL_93012* was unaffected in the *efg1Δ* strain.

Overexpression of *O*. *polymorpha EFG1* also caused changes of expression of some white-opaque genes. *CZF1-like2* was down-regulated 20-fold, and *CZF1-like3* was up-regulated 7-fold (Fig 3C; S5A Table). *WOR3* and *WOR4*, which are more recently identified components of the white-opaque circuit in *C*. *albicans* [43, 44], were down-regulated (10- and 12-fold respectively). The regulatory relationship between *EFG1* and *CZF1-like2* appears to be complex, because *CZF1-like2* was down-regulated by both deletion and overexpression of *EFG1*.

### *RME1* and *STE12*, as well as *EFG1*, are necessary for mating-type switching and mating

Based on the results of the *EFG1* deletion and overexpression mRNAseq analyses, we tested whether *RME1* and the *EFG1* paralog *OPOL_93012* are required for mating-type switching and/or mating. We also tested *STE12*, which plays a central role in the mating response in other yeast species, and which shows induction by nitrogen depletion (Fig 3C; S1 Table). *MAT***a** and *MAT*α deletion strains for each gene were constructed and tested for their ability to switch mating types, and to mate.

**Fig 4:**
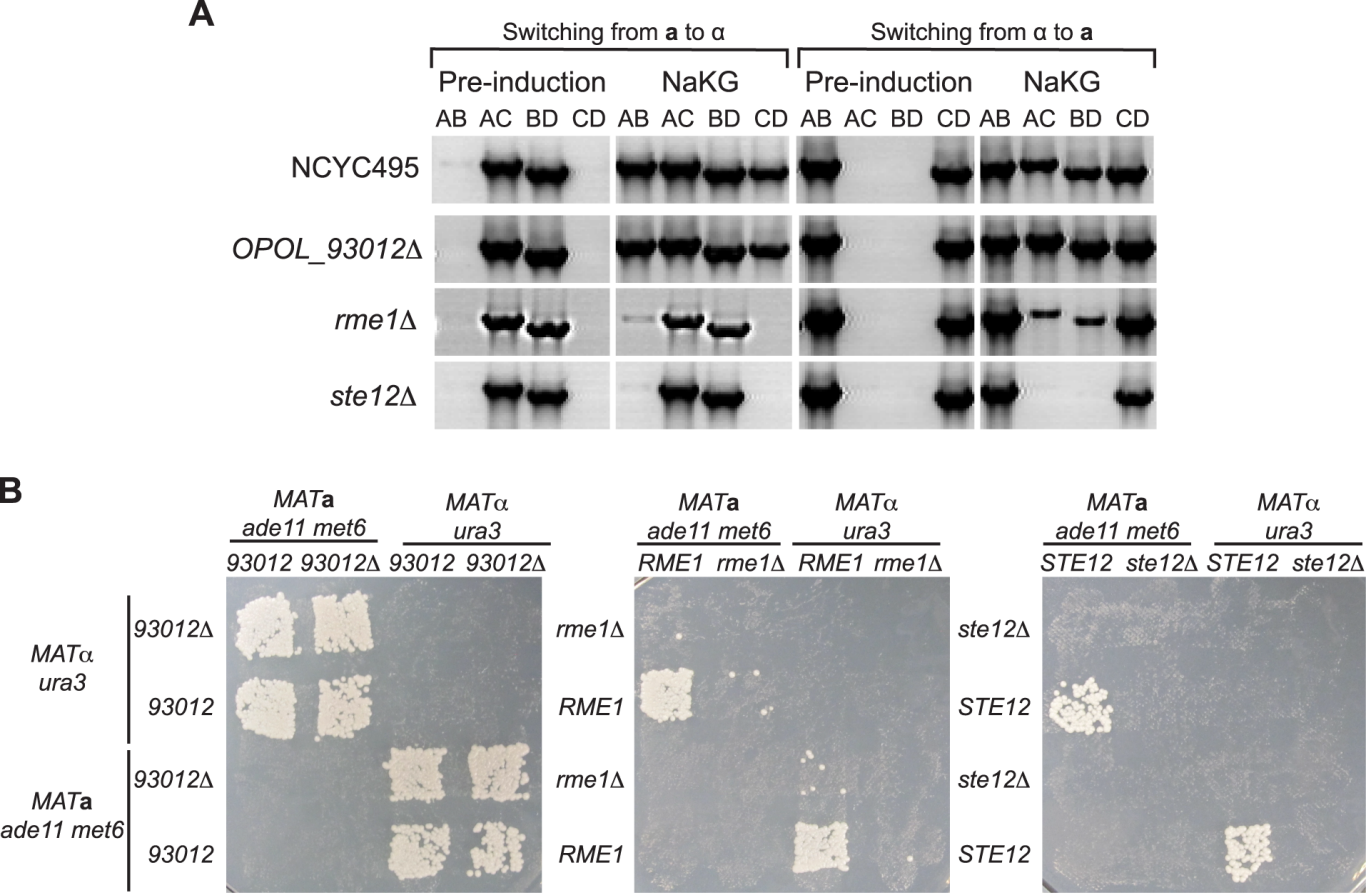
*RME1* and *STE12* are required for *O*. *polymorpha* mating-type switching and mating, but *OPOL_93012* is not. **(A)** PCR determination of *MAT* genotypes before and after 24 h growth in NaKG of *opol_93012Δ*, *rme1Δ*, and *ste12Δ* strains. Deletion strains were constructed in both *MAT***a** and *MAT*α backgrounds. The panels for the wild-type NCYC495 controls are reproduced from Fig 3A because the two experiments were carried out together. PCR primers A-D are as in Fig 1. **(B)** Photographs of diploid growth on SD plates following crosses of haploids on MEMA for 24 h.

Deletion of *RME1* severely reduced mating-type switching, as measured by PCR assay, in both *MAT***a** and *MAT*α cells (Fig 4A), similar to the result from *EFG1* deletion (Fig 3A). Deletion of *STE12* completely abolished switching. Furthermore, deletion of *RME1* or *STE12* abolished mating, in crosses where both parents were *rme1Δ* or *ste12Δ* (Fig 4B). Crossing *rme1Δ* x *RME1* resulted in a low number of diploid colonies, similar to *efg1Δ* x *EFG1* crosses, whereas *ste12Δ* x *STE12* crosses did not produce any colonies (Fig 4B). In contrast to these three genes, deleting the *EFG1* paralog *OPOL_93012* had no effect on switching or mating (Fig 4A and 4B).

Because *EFG1* is a component of the white-opaque circuit in *C*. *albicans*, and because several *O*. *polymorpha* homologs of white-opaque genes were found to be differentially regulated in our transcriptome analyses as mentioned above (Fig 3C), we also made deletion strains of four *O*. *polymorpha* ‘white-opaque’ genes: *CZF1-like2*, *CZF1-like3*, *MIT1 (WOR1)*, and *WOR2* (S7 Fig). However, none of these deletions had any effect on either switching or mating (S7 Fig).

### Overexpression of *RME1* or *STE12*, but not *EFG1*, is sufficient to induce switching without an environmental signal

**Fig 5:**
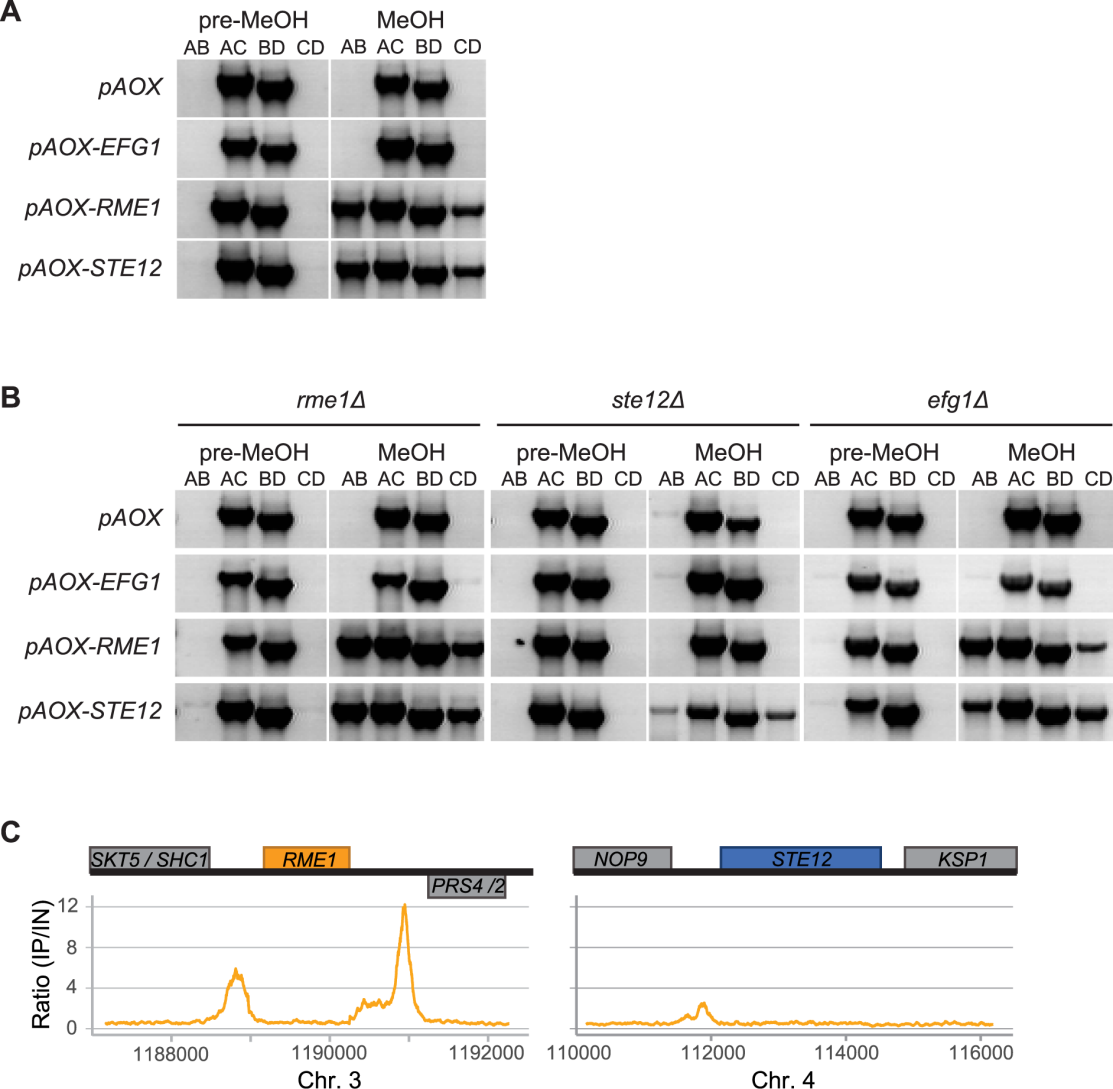
*RME1* induces switching in *O*. *polymorpha* through activation of *STE12*. **(A,B)** PCR determination of *MAT* genotypes before and after induction of *pAOX* expression by growth in methanol (MeOH) in (A) wild-type and (B) knockout backgrounds. All strains initially had *MAT***a** genotype. **(C)** *RME1* binds to its own promoter and 3’ region, and to the *STE12* promoter. The Y-axis represents the ratio of immunoprecipitated to input reads from ChIPseq of 3xHA-tagged Rme1 protein across the *RME1* and *STE12* loci in the *O*. *polymorpha* genome. Genes transcribed rightwards are drawn above the horizontal line, and genes transcribed leftwards are below it.

Since *EFG1*, *RME1* and *STE12* are all necessary for switching, we investigated whether high expression of any of them is also sufficient to induce switching, even in the absence of a nitrogen depletion signal. We constructed methanol-inducible *pAOX-RME1* and *pAOX-STE12* strains similar to the *pAOX-EFG1* strain described above. Switching was induced when strains containing *pAOX-RME1* or *pAOX-STE12* were transferred from glucose to methanol (Fig 5A), demonstrating that overexpressed *RME1* and *STE12* are each sufficient to induce switching. The *pAOX-EFG1* strain, and a control strain containing the *pAOX* vector alone, did not switch under the same conditions (Fig 5A). The latter result is consistent with the observation that *EFG1* is transcribed in nitrogen-rich as well as nitrogen-poor conditions, and indicates that *EFG1* requires additional factors in order to induce switching.

### *RME1* acts upstream of *STE12*

The results we have presented so far show that *EFG1*, *RME1* and *STE12* are each necessary for switching, and that *EFG1* acts upstream of *RME1*. To determine where *STE12* fits into the pathway, we constructed strains that combined overexpression of one gene with deletion of another. We introduced the *pAOX-RME1*, *pAOX-STE12*, and *pAOX-EFG1* constructs individually into the deletion strains *rme1Δ*, *ste12Δ*, and *efg1Δ* in all possible combinations (Fig 5B). Methanol induction of *EFG1* was again unable to induce switching in any background, whereas *STE12* overexpression induced switching in all backgrounds. *RME1* overexpression, although sufficient for switching in the *rme1Δ* and *efg1Δ* backgrounds, did not induce switching in the *ste12Δ* strain. This result indicates that *RME1* acts upstream of *STE12* in the switching pathway.

To test whether *RME1* binds to the promoter of *STE12*, we performed ChIPseq using 3xHA-tagged Rme1, expressed from its native chromosomal locus. In addition to binding to its own promoter and 3’ UTR, Rme1 bound to the promoter of *STE12* (Fig 5C). Furthermore, mRNAseq analysis of the *pAOX-RME1* strain shows that overexpression of *RME1* results in an increase in expression of *STE12* (S8 Fig; S5C Table). Together, these data suggest that *RME1* directly activates transcription of *STE12* by binding to its promoter, which leads to switching.

### *RME1* and *STE12* overexpression induces the mating pathway including pheromone genes

Since overexpression of either *RME1* or *STE12* induces switching, we tried to identify components further downstream in the switching pathway by transcriptome analysis of the *pAOX-RME1* and *pAOX-STE12* overexpression strains, after overnight growth in methanol media. We found that the major consequence of overexpressing these transcription factors was strong induction of genes in the mating response pathway (S8 Fig; S5 Table), consistent with the essential roles of *RME1* and *STE12* in *O*. *polymorpha* mating (Fig 4). The genes induced included orthologs of the *S*. *cerevisiae* haploid-specific genes (*STE4*, *GPA1*, *STE18*, *STE5*, *FAR1*, *FUS3*) required for transmission of the pheromone signal. Our *pAOX* overexpression strains were constructed in a haploid *MAT***a** background, and we detected methanol-induced transcription of **a**-specific genes (*BAR1*, *AXL1*, *ASG7*, *RAM1*, *RAM2*, *STE6*) that are required for production of **a**-factor and modulation of the α-factor signal. The mating pathway induction by *STE12* overexpression was so strong that it enabled us to annotate the **a**-factor gene (*MF***a**) of *O*. *polymorpha* for the first time (S9 Fig). We also observed induction of the α-specific genes *MAT*α1, *MAT*α2 and the α-factor gene *MF*α, which is likely due to expression in cells that had successfully switched mating-type from *MAT***a** to *MAT*α in the cultures (Fig 5A). In addition to the mating pathway genes, overexpression of *RME1* (but not *STE12*) also induced transcription of genes with roles in sporulation such as *RIM4*, *IME2*, *MUM2*, and *MEI2* (S5C Table). In contrast to *RME1* and *STE12*, *EFG1* overexpression did not significantly induce expression of mating pathway genes (S8 Fig).

Disappointingly, the *RME1* and *STE12* overexpression mRNAseq analyses did not reveal any clear candidates for genes that act downstream in the switching pathway. They did however show that *RME1* and *STE12* form a positive feedback loop. Overexpression of *RME1* induced *STE12* by 14-fold, and overexpression of *STE12* induced *RME1* by 9-fold (S5B Table, S5C Table). They also showed that overexpression of *RME1* induced expression of *CZF1-like3* and repressed expression of *CZF1-like2* (S5A Table), similar to overexpression of *EFG1*, so the effect of *EFG1* on these white-opaque genes is probably mediated through *RME1*.

### The homologous recombination pathway is required for mating-type switching

Although the transcriptomic and ChIPseq experiments did not identify obvious candidates for downstream roles in the switching pathway, such as homologs of known DNA recombinases or endonucleases, they did uncover several *O*. *polymorpha* genes of unknown function whose patterns of transcription were consistent with the profile we expected switching pathway genes to have. We chose 21 candidate *O*. *polymorpha* genes for deletion and testing of switching phenotypes, including (i) genes of unknown function with appropriate transcription profiles, (ii) orthologs of *S*. *cerevisiae* genes that interact with *STE12*, such as *TEC1*, *FUS3* and *KSS1*, and (iii) orthologs of *S*. *cerevisiae* genes with roles in mating-type switching, homologous recombination or DNA repair, such as *ASH1*, *RAD51* and *PMS1*. Deletion strains of each of the 21 genes in an NCYC495 *ku80Δ MAT*α background were tested for their ability to switch mating-types, of which 19 had no phenotype (S10 Fig).

**Fig 6:**
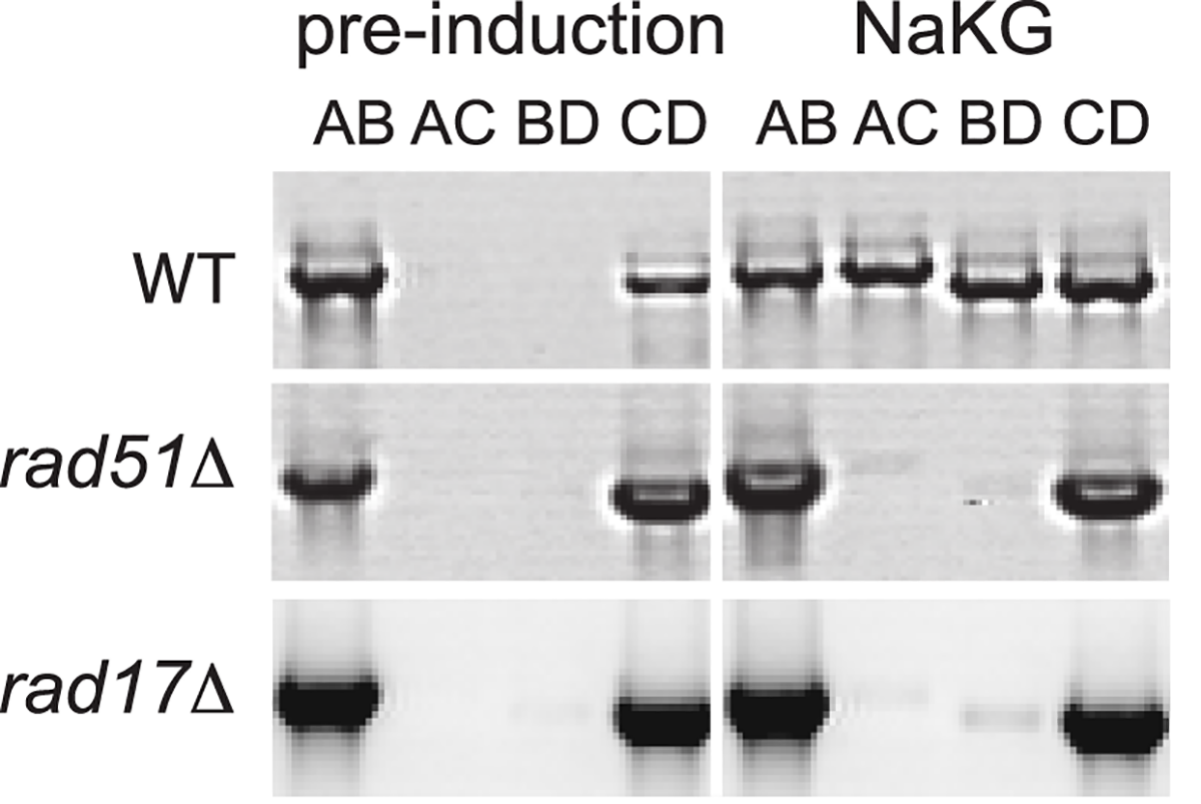
*RAD51* and *RAD17* are required for mating-type switching. Deletions were constructed in a *MAT*α *ku80Δ* background. The *MAT* locus was PCR amplified from each strain before, and 24 h after, transfer from a YPD pre-induction culture into NaKG. The wildtype (WT, NCYC495 *ku80Δ*) panels in this figure are reproduced from Fig 1C because these experiments were done together.

We found that mating-type switching was almost completely abolished in strains with deletions of the orthologs of two *S*. *cerevisiae* genes in the homologous recombination pathway, *RAD51* and *RAD17* (Fig 6). In *S*. *cerevisiae*, Rad51 is a single-stranded DNA binding protein that mediates strand exchange during homologous recombination [45], and is necessary for mating-type switching [46]. The *O*. *polymorpha RAD51* gene has previously been reported to partially complement an *S*. *cerevisiae rad51* mutant, and the protein catalyzes DNA strand exchange *in vitro* [47]. Rad17 is a component of the checkpoint signaling clamp called 9-1-1 in humans or Ddc1-Mec3-Rad17 in *S*. *cerevisiae* [45]. The Mec3 component of the clamp is not necessary for mating-type switching in *S*. *cerevisiae* [48], but whether Rad17 is necessary has not been investigated. The requirement for *RAD51* and *RAD17* in *O*. *polymorpha* switching shows that the homologous recombination pathway for repair of DNA breaks is involved in the interaction between the IRs.

## Discussion

**Fig 7:**
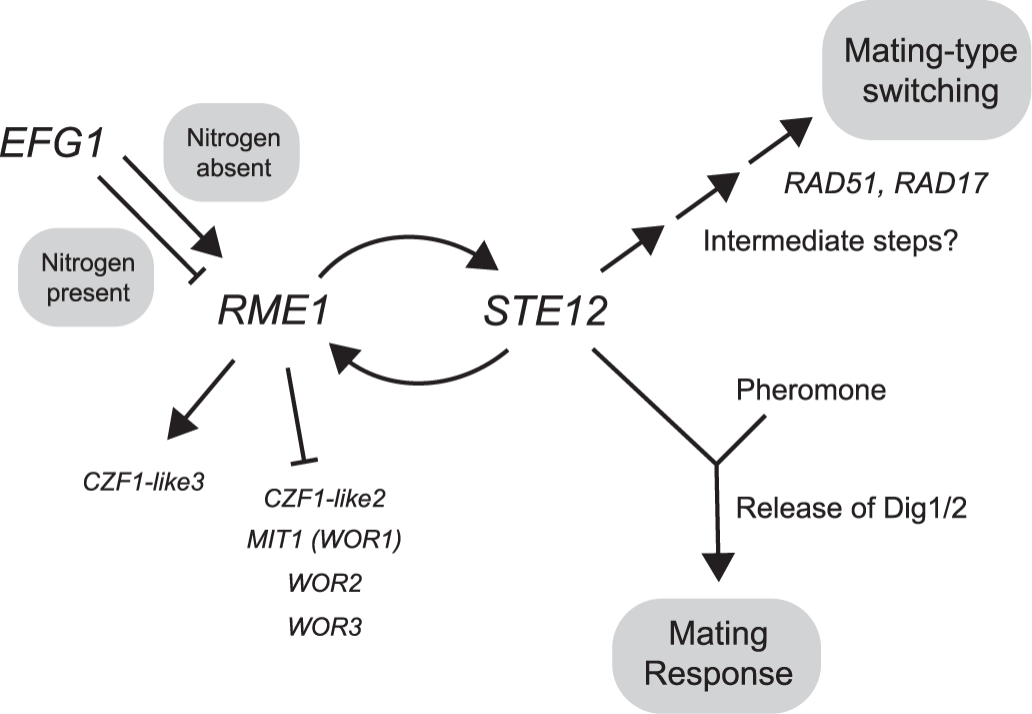
Model for regulation of mating-type switching and the mating response in *O*. *polymorpha*.

Our experimental results suggest a model for how mating-type switching and the mating response to pheromone are both controlled in in *O*. *polymorpha* (Fig 7). In the presence of a nitrogen source, an *EFG1*-dependent mechanism represses transcription of *RME1* and the whole pathway is inactive. In the absence of a nitrogen source, *RME1* is active and a positive feedback loop between *RME1* and *STE12* expression develops. If pheromone is detected, *STE12* activates the mating response pathway and mating ensues. We postulate that if no pheromone is detected, *STE12* instead activates mating-type switching, which could then lead to mating with a cell of the original mating-type. The final steps in switching utilize the homologous recombination pathway, but the intermediate steps connecting *STE12* to the *RAD* genes remain unknown.

As shown in Fig 7, *RME1* also has a role in regulating ‘white-opaque’ transcription factors. We do not know if a regulatory loop similar to the *C*. *albicans* white-opaque circuit exists in *O*. *polymorpha*, but in any case our gene deletion experiments (S7 Fig) show that white-opaque genes other than *EFG1* have no role in switching or mating. In *C*. *albicans*, *EFG1* is the main activator of the mating-incompetent (white) state [40], whereas in *O*. *polymorpha EFG1* is required for mating competence. Our model may be oversimplified because the connection between *EFG1* and *RME1* seems to be complex and may involve intermediate steps. By analysis of the *efg1Δ* strain we found that in nitrogen-rich conditions *EFG1* causes repression of *RME1*, whereas in nitrogen-poor conditions *EFG1* was essential for switching and mating, suggesting conversely that it causes activation of *RME1*. Since overexpression of *EFG1* had no effect on *RME1* transcription, the activity of *EFG1* may depend on other factors such as the presence of partner proteins, or post-translational modification of Efg1. The Efg1 proteins of some yeast species are known to be phosphorylated [49–51]. *C*. *albicans* Efg1 can act as both a repressor and an activator [26, 52], so it is possible that *O*. *polymorpha* Efg1 can both positively and negatively affect *RME1* transcription in different conditions.

A fundamental difference between *O*. *polymorpha* and *S*. *cerevisiae* is that in *S*. *cerevisiae*, detection of pheromone is the only signal necessary to trigger a mating response, whereas in *O*. *polymorpha* a nitrogen depletion signal is needed as well. Ste12 is the probable point of integration of these two signals in *O*. *polymorpha* (Fig 7), with the nitrogen-depletion signal (communicated through Efg1 and Rme1) increasing the level of *STE12* transcription, and the pheromone-induced MAP kinase cascade activating Ste12 by releasing the ortholog of the inhibitor proteins Dig1/Dig2 [53, 54]. We suggest that *O*. *polymorpha* cells initiate switching if Ste12 protein becomes abundant but no pheromone has been detected.

**Fig 8:**
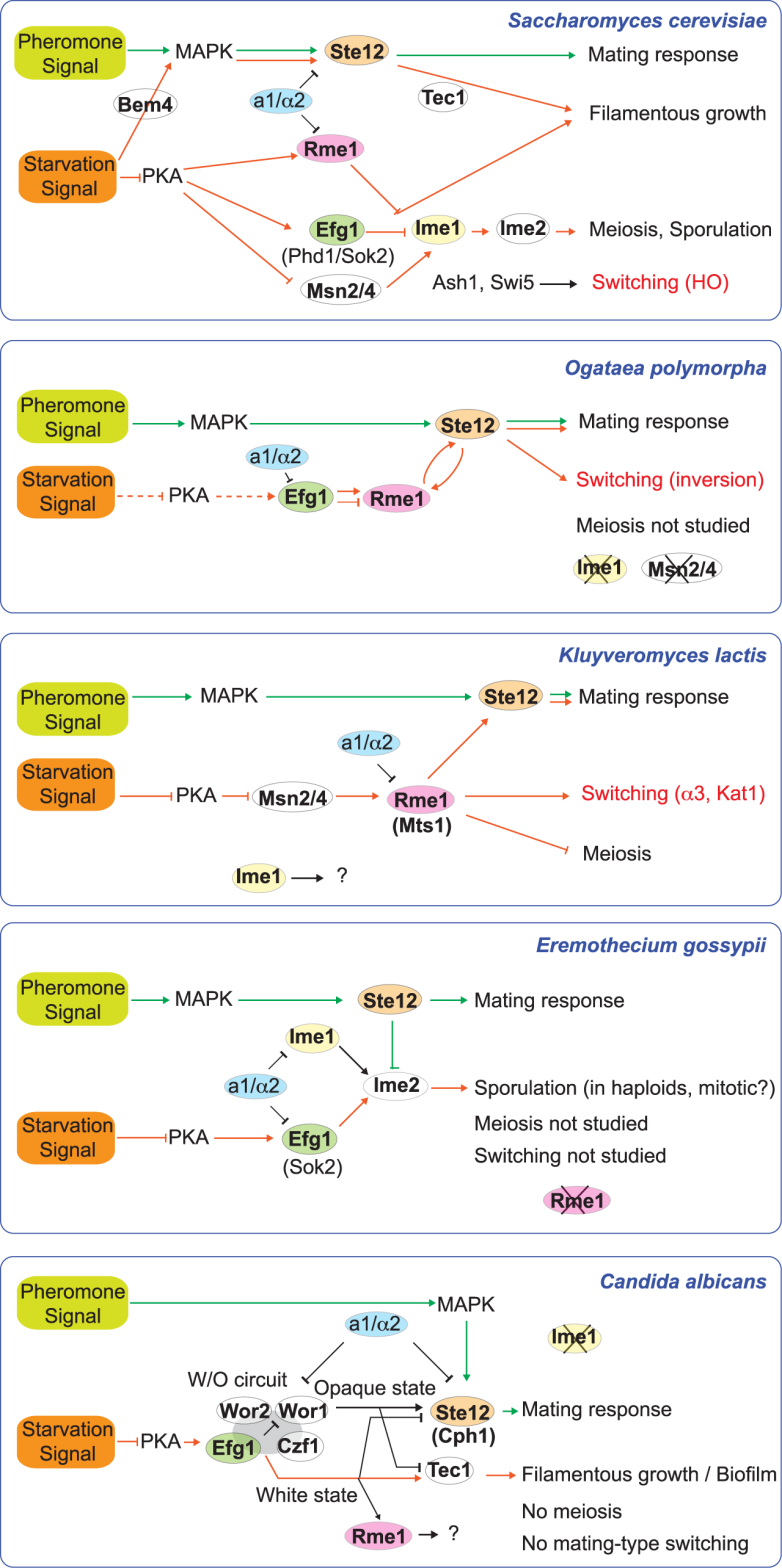
Comparison of regulatory networks in five ascomycete species. Orange and green arrows refer to communication of nutrient depletion and pheromone signals, respectively. Pathways are summarized from the literature for *S*. *cerevisiae* [1, 20, 38, 49, 53, 56, 68–70], *K*. *lactis* [15, 37, 61], *E*. *gossypii* [59], and *C*. *albicans* [26, 40, 54, 60, 71, 72]. *C*. *albicans* has an *RME1* ortholog whose transcription is specific to the white state [73]. It has no role in mating [37] and its function is unknown.

Comparing the networks that contain *EFG1*, *RME1* and *STE12* in different ascomycete species shows that there has been extensive reorganization during evolution (Fig 8). These networks are complex because they integrate information about the cell’s nutrient status (from the PKA pathway), the presence of pheromone (from the MAPK pathway), and the cell’s ploidy (from the **a**1/α2 repressor), to decide whether the cell responds by mating, switching, sporulating, or filamentous growth [55]. In *S*. *cerevisiae*, the nutrient status of the cell is primarily signaled by modulating PKA activity, which occurs via cyclic AMP for glucose sensing, and independently of cAMP for sensing other nutrients such as nitrogen [36, 56]. Much of the transcriptional response to changes in PKA activity in *S*. *cerevisiae* is mediated by the stress-response transcription factors Msn2 and Msn4 [56, 57]. However, there is no ortholog of Msn2/4 in *O*. *polymorpha*, and in *C*. *albicans* the major PKA-regulated transcription factor is Efg1, not Msn2/4 [33–36, 58]. It is likely that in *O*. *polymorpha* PKA regulates *EFG1* to signal nitrogen depletion, because PKA is known to regulate Efg1 orthologs in *Eremothecium (Ashbya) gossypii* [59] and *S*. *cerevisiae* [28, 57], as well as *C*. *albicans* [51, 60]. One of the functions of *S*. *cerevisiae* Sok2 is to repress the master inducer of meiosis *IME1* in rich conditions [49], but *IME1* also has no ortholog in *O*. *polymorpha*. Thus the role of *O*. *polymorpha EFG1* may be quite unlike the roles of *S*. *cerevisiae SOK2* and *PHD1*. The MAPK and PKA pathways may be more interconnected in other ascomycetes than in *S*. *cerevisiae*, because *C*. *albicans* Efg1 also plays a role in mating and interacts with the Dig1/2 ortholog [54].

The mating response pathway of *O*. *polymorpha* is similar to that in *K*. *lactis* in the sense that Rme1 conveys the nutrient depletion signal that is needed to activate *STE12* for mating [20, 37], but their pathways for induction of switching are different (Fig 8). In *K*. *lactis*, Ste12 has no known role in switching, and upon nutrient depletion Rme1 induces switching either by activating transcription of *KAT1* or by binding to the α3 locus, depending on the direction of switching [14, 15]. Furthermore, the connection between nutrient signaling and switching in *K*. *lactis* has been proposed to occur via Msn2 rather than Efg1 [61]. Nevertheless, the pathway that regulates switching in *O*. *polymorpha* has more similarity to that in *K*. *lactis* than to that in *S*. *cerevisiae*. Switching in *S*. *cerevisiae* via HO endonuclease is highly regulated in terms of cell cycle and cell lineage [3, 4, 13], but has no connection to PKA signaling or *STE12* (Fig 8).

Is there an endonuclease or site-specific recombinase for mating-type switching in *O*. *polymorpha*? At the outset of this project we assumed that the flip/flop mechanism would employ a specific enzyme to initiate recombination between the two IRs, but we have been unable to find such an enzyme. In retrospect, we realize that a site-specific recombinase is unlikely because recombinases generally recognize sites that are much shorter than the 2-kb IRs [62]. It now seems probable that during switching a site-specific DNA break is induced in one copy of the IR, followed by repair by recombination with the other copy, which can be resolved as either a crossover (inversion of the 19-kb region) or a non-crossover (no switching). Site-specific breaks are made in *S*. *cerevisiae* by HO, and in *K*. *lactis* by Kat1 and α3, but the *O*. *polymorpha* genome contains no homologs of any of these proteins. One possible hypothesis for *O*. *polymorpha* is that a site-specific break might be formed during attempted replication of a fragile DNA site, similar to switching in *Schizosaccharomyces pombe* [4, 9], but if this is correct, the site must be fragile only in nitrogen-poor conditions. Alternatively, *O*. *polymorpha* might use a recombinase or endonuclease that is activated post-transcriptionally. Further characterization of the switching mechanism in *O*. *polymorpha* may require biochemical approaches or genetic screens to identify mutants that switch constitutively.

## Materials and methods

### Strain and plasmids

Strains and plasmids used in this study are listed in S6 Table. Constructs for gene deletions contained 700–1000 base pairs of sequence flanking the target locus and an antibiotic resistance marker. Flanking and marker sequences were amplified using a high-fidelity DNA polymerase (Phusion or Q5, New England Biolabs), purified (PCR Purification Kit, Qiagen), and assembled by fusion PCR. PCR products were introduced into cells by electrotransformation, as described previously [63]. Gene deletions were made in *ku80Δ* backgrounds to increase efficiency of homologous recombination [32]. Successful integration was tested by antibiotic selection on YPD plates containing 200 μg/mL G418, 200 μg/mL hygromycin B, 100 μg/mL nourseothricin, or 100 μg/mL zeocin, as appropriate. Colony PCR was performed on resistant colonies to test for integration at the correct locus (GoTaq G2 polymerase, Promega). Plasmids for overexpression were constructed using pHIPH4 [32]. *EFG1*, *STE12*, and *RME1* coding sequences were amplified using a high fidelity polymerase and primers containing restriction enzyme sites (SbfI, XmaI, HindIII, or XbaI). The purified PCR products and plasmid were digested, ligated, and transformed into *E*. *coli*. Clones were purified and digested with StuI enzyme overnight at 37°C for electrotransformation into *O*. *polymorpha*.

### Mating-type switching PCR assay

To induce mating-type switching, overnight ‘pre-induction’ cultures grown at 37°C in YPD were centrifuged at 3400 x *g* for 2 min, washed once in NaKG (0.5% sodium acetate, 1% potassium chloride, 1% glucose), and resuspended in 10 mL NaKG at A_600_ 0.5. NaKG cultures were then incubated on a shaker at 28°C for 24 h. DNA was isolated from the pre-incubation and NaKG-grown cultures by phenol:chloroform extraction. The *MAT* locus orientation was determined by PCR amplification using GoTaq G2 polymerase (Promega) for 30 cycles with 55°C annealing temperature and 3 min elongation. PCR products were visualized on 1% agarose gel with ethidium bromide staining.

### Mating assay

Cells were streaked in parallel lines on YPD agar and crossed on MEMA (2.5% maltose, 0.5% malt extract, 2% agar) by replica plating. MEMA plates were incubated at 28°C for 24 h before replica plating to SD agar. SD plates were incubated at 37°C for 48 h to observe growth of diploids.

### Methanol induction

Induction of expression from the *AOX* promoter was achieved by growing overnight cultures at 37°C in mineral media [64] containing 0.5% glucose (MMG). Overnight cultures were diluted in fresh MMG to A_600_ 0.2 and grown to A_600_ >1.5. Cultures were diluted again in fresh MMG to A_600_ 0.2 and grown to A_600_ >2.0. Cultures were diluted in mineral media + 0.4% methanol (MMM) to A_600_ 0.2 and grown on shaker overnight at 37°C. RNA samples were isolated from these cultures with two biological replicates by hot acid phenol extraction and DNase I (Invitrogen) treatment.

### Bulk segregant analysis

A diploid prototrophic colony obtained from the *O*. *polymorpha* NCYC495 x *O*. *parapolymorpha* DL-1 interspecies cross was sporulated by streaking on ME agar (2% malt extract, 2% agar) and incubating at 25°C. Random spores were isolated by ether treatment: sporulating culture was suspended in sterile water before addition of an equal volume of diethyl ether and incubation at 30°C for 45 min. Ether-treated cells were diluted, plated on YPD agar, and incubated at 37°C for 48 h. Haploid clones grown from spores were tested for the ability to switch mating types using the PCR assay described above, with the following modification: DNA extractions were performed by treatment of cells with 700 units lyticase, incubation at 37°C for 30 min, followed by extraction using a Promega Maxwell 16 according to manufacturer’s instructions. Clones were identified as *MAT***a** or *MAT*α, and as switchers or non-switchers, by PCR assay. Clones with clear phenotypes were assigned to four pools for sequencing: *MAT*α switchers (30 clones), *MAT*α non-switchers (35 clones), *MAT***a** switchers (35 clones) and *MAT***a** non-switchers (52 clones) (S5A Fig). Clones for each pool were grown individually and the pools were then made by combining equal A_600_ units for phenol:chloroform DNA extraction. DNA was also extracted from the parental strains NCYC495 and DL-1. All DNA samples were purified using a Genomic DNA Clean and Concentrator kit (Zymo). Genomic DNA library preparation and Illumina HiSeq 2500 sequencing were performed at the University of Missouri DNA Core Facility.

We first created new reference genome sequences for our *O*. *polymorpha* NCYC495 (*ade11 met6*) and *O*. *parapolymorpha* DL-1 (*leu2 ura3*) parental strains, by mapping the reads from these strains onto the published genome sequences [12, 24] using BWA [65]. We did this because we discovered that our *ade11 met6* derivative of NCYC495 (obtained from Dr. Kantcho Lahtchev, Bulgarian Academy of Sciences) contains regions with significant numbers of differences relative to the reference sequence of strain NCYC495 *leu1*.*1* (obtained from Prof. Andriy Sibirny, National Academy of Sciences of Ukraine) that was sequenced by Riley et al. [12]. Our ‘NCYC495’ *ade11 met6* stock appears to be the product of a cross between a genuine NCYC495 background and an *O*. *polymorpha* strain with a slightly divergent genome, possibly strain CBS4732.

We then mapped the Illumina reads from each of the four pools to these *O*. *polymorpha* and *O*. *parapolymorpha* reference genome sequences. Only reads that had a single perfect match to one species, but no perfect match to the other, were retained for analysis. We divided the *Ogataea* genome into 7824 segments, where each segment is either a pair of orthologous genes in NCYC495 and DL-1, or a pair of ‘intergenic’ regions in the interval between two consecutive pairs of orthologs. These ‘intergenic’ regions can include genes that are present in one species but absent in the other. For each segment in each species, we calculated the numbers of reads from each pool, and from the parental strains, that mapped to it. Preliminary analysis showed no significant differences between the two mating types, so we merged the data from *MAT***a** and *MAT*α clones. We then calculated four ratios for each genomic segment:

- *SW*_NCYC495_ is the number of reads from switchers that mapped exclusively to the NCYC495 reference genome in this segment, divided by the number of reads from the NCYC495 parental strain that mapped exclusively to the NCYC495 reference genome in this segment, normalized by the total numbers of mapped reads in each library.
- *NS*_NCYC495_ is the number of reads from non-switchers that mapped exclusively to the NCYC495 reference genome in this segment, divided by the number of reads from the NCYC495 parental strain that mapped exclusively to the NCYC495 reference genome in this segment, normalized by the total numbers of mapped reads in each library.
- *SW*_DL-1_ is the number of reads from switchers that mapped exclusively to the DL-1 reference genome in this segment, divided by the number of reads from the DL-1 parental strain that mapped exclusively to the DL-1 reference genome in this segment, normalized by the total numbers of mapped reads in each library.
- *NS*_DL-1_ is the number of reads from non-switchers that mapped exclusively to the DL-1 reference genome in this segment, divided by the number of reads from the DL-1 parental strain that mapped exclusively to the DL-1 reference genome in this segment, normalized by the total numbers of mapped reads in each library.

These four ratios are plotted in S5B Fig. We defined the Asymmetry metric (Fig 2C) of a genomic segment as

$$\mathrm{Asymmetry}=\max\left(SW_{\mathrm{NCYC}495}\;\text{-}\;1,0\right)\;*\;\max\left(1\;\text{-}\;NS_{\mathrm{NCYC}495},0\right)\;*\;\max\left(NS_{\mathrm{DL}\text{-}1}\text{-}1,0\right)\;*\;\max\left(1\;\text{-}\;SW_{\mathrm{DL}\text{-}1},0\right)$$

This metric has a value of zero, except in genomic segments where four criteria are met simultaneously: the proportion of NCYC495-derived DNA is higher than expected by chance in the switcher pool but lower than expected in the non-switcher pool, and the proportion of DL-1-derived DNA is higher than expected by chance in the non-switcher pool but lower than expected in the switcher pool.

### RNAseq and ChIPseq

Strains for nitrogen depletion samples and *efg1* deletion samples were grown in YPD at 37°C overnight, diluted to an OD_600_ 0.1 and grown to log phase (OD_600_ 1.0). Cultures were pelleted by centrifugation, washed once in YPD, NaKG, NaKG + 40mM ammonium sulfate, SD, or SD minus ammonium sulfate, before resuspending in the same media and culturing for 2 h at 28–30°C. RNA samples from these cultures were prepared with the MasterPure Yeast RNA Purification Kit (Epicentre, Illumina) or by hot acid phenol extraction and DNase I (Invitrogen) treatment. Nitrogen depletion samples were performed in triplicate, overexpression and *efg1* deletion samples were performed in duplicate.

Chromatin immunoprecipitation (ChIP) was performed by formaldehyde crosslinking of log phase cells for 20 min with glycine addition used to stop the reaction. Cells were lysed using glass beads and chromatin was fragmented by sonication with a Bioruptor Standard (Diagenode). EZview Red Anti-HA Affinity Gel (Sigma-Aldrich) was used to immunoprecipitate chromatin fragments, and bound DNA was eluted using HA peptide (Sigma-Aldrich). Crosslink reversal was followed by phenol:chloroform extraction. ChIP samples were performed with three biological replicates that were pooled prior to sequencing.

Stranded mRNAseq and ChIPseq library preparation and sequencing services were performed at the University of Missouri DNA Core Facility. 50–51 bp unpaired Illumina reads (RNAseq and ChIPseq) were mapped to the *Ogataea polymorpha* (NCYC495 *leu1*.*1* [12]) genome using Bowtie v1.1.2 using the following options: -v = 3, to report end-to-end hits with < = 3 mismatches; -k = 10, to report up to 10 good alignments per read;—best, so hits guaranteed best stratum with ties broken by quality; -M = 1, to report just 1 random hit out of the good alignments for a read; -S, to write hits in SAM format; -p = 10, to use 10 processors. Aligned hits were split into reads that mapped to the forward and reverse strand (SAM FLAG = 0 and 16) before proceeding. Samtools v0.1.12a (r862) was used to create sorted and indexed BAM files of the results. Bedtools v2.19.0 was used to create genome coverage Bedgraph files, which were converted to BigWig files using bedGraphToBigWig v4 for visualization as tracks in Jbrowse v1.11.2.

For RNAseq data htseq-count v0.6.0 was used to calculate for each feature the number of reads mapping to it. We mapped to a feature file based on the original JGI NCYC495 annotation with extensive manual modification. We counted against both the forward and reverse strand mapping SAM files, creating sense and antisense counts for each feature, but only retained sense counts for further analysis. We then calculated Transcripts Per Million (TPMs) [66] for each feature and used DESeq2 in R v3.2.1 to calculate differential expression between conditions.

## Supporting information

S1 Fig Cell-type and media dependent gene expression patterns in *O*. *polymorpha*. Heatmap shows the log_2_(fold change) in expression of all genes in the genome from mRNAseq of *O*. *polymorpha* haploid *MAT***a**, haploid *MAT*α, and diploid *MAT***a**/α strains grown in rich media (YPD), or 2 h after transfer from YPD into nitrogen depletion media (NaKG).
(EPS)

S2 Fig Gene expression differences between a-cells and α-cells. Each gene’s expression ratio between **a**-cells and α-cells is plotted, for NaKG media (X-axis) and YPD media (Y-axis). Only the 475 genes for which the expression ratio was significantly different from 1 in at least one of the conditions are plotted (unadjusted *P* < 1e-3).
(EPS)

S3 Fig Gene expression differences between haploid a and diploid a/α *O*. *polymorpha* cells. For each gene in the genome, its expression ratio between **a**-cells and **a**/α-cells is plotted, in NaKG media (X-axis) and YPD media (Y-axis). The *MAT*α1 and *MAT*α2 genes appear diploid-enriched in this experiment because they have higher expression in diploid **a**/α-cells than in haploid **a**-cells. Only genes for which the expression difference was significant at P < 1e-3 (adjusted for multiple testing with the Benjamini-Hochberg correction) for at least one of the media are plotted.
(EPS)

S4 Fig Search for haploid-specific, NaKG-induced genes. Environmental control of gene expression in haploid cells (X-axis) is compared to cell type control of gene expression in nitrogen limitation media (Y-axis). Genes with a role in mating-type switching are expected to lie in the bottom-left quadrant.
(EPS)

S5 Fig **(A)** Phenotypes of haploids used in bulk segregant analysis. Random spores isolated from a DL-1 x NCYC495 diploid were grown overnight in YPD before transfer to NaKG for 24 h. Gels show PCR analysis of the *MAT* locus to determine the original mating type and switching phenotype of the haploid isolates. Haploids were classified into four groups as shown. **(B)** Inheritance of genomic regions derived from NCYC495 and DL-1 in the sequenced pools of switching and non-switching progeny. The upper panel shows reads that mapped exclusively to the NCYC495 reference genome, and the lower panel shows reads that mapped exclusively to the DL-1 reference genome, from switcher (blue) and non-switcher (red) pools. The Y-axis is the ratio between the normalized number of mapped reads from a pool, relative to the number from the parental strain sample, in each of the 7824 genomic segments. Chromosome numbering and orientation follows the convention for *O*. *polymorpha* [12].
(TIF)

S6 Fig **(A)** Multiple sequence alignment of Efg1 and related proteins. The APSES domain is highlighted in blue. The orange triangle indicates the site of the frameshift mutation in *O*. *parapolymorpha* strain DL-1. **(B)** Phylogenetic tree of the Efg1 protein family. *O*. *polymorpha* has two *EFG1-*like genes, which we refer to as *EFG1* (*OPOL_95241*) and *OPOL_93012*. This gene duplication is specific to the genus *Ogataea*, shared by *O*. *polymorpha* and *O*. *parapolymorpha*. The tree was constructed by Maximum Likelihood using an LG model with an alignment of 1377 amino acids in 55 taxa from Saccharomycotina and Pezizomycotina. Branch support was determined by 100 bootstrap replicates.
(EPS)

S7 Fig White-opaque circuit genes other than *EFG1* are not required for mating-type switching or mating in *O*. *polymorpha*. **(A)** PCR determination of *MAT* genotypes before and after 24 h growth in NaKG for wild-type, *czf1-like2*Δ, *czf1-like3*Δ, *czf1-like2*Δ*czf1-like3*Δ, *mit1*Δ and *wor2*Δ *O*. *polymorpha* strains in *MAT***a** (left) and *MAT*α (right) backgrounds. PCR primers A-D are as in Fig 1. (B) Photographs of diploid growth on SD plates following crosses on MEMA plates for 24 h.
(EPS)

S8 Fig *RME1* and *STE12* overexpression induces the mating pathway in *O*. *polymorpha*. **(Left)** Heatmap showing the log_2_(fold change) of expression of all genes in mRNAseq of strains overexpressing *RME1*, *STE12*, or *EFG1* from the *pAOX* methanol-inducible promoter, relative to a control strain containing empty *pAOX* vector. **(Right)** Genes with the highest expression increases in *STE12* and *RME1* overexpression strains include those with **a**-specific (gene names in green), α-specific (pink), haploid-specific (purple), and other mating functions (orange). Genes with Rme1 signals in ChIPseq are indicated by orange boxes.
(EPS)

S9 Fig Alignment of a-factor (MFa) sequences from *O*. *polymorpha*, *S*. *cerevisiae* and *C*. *albicans*. The *O*. *polymorpha* gene is located immediately upstream of *RAD17* on chromosome 5, at position complement(253651..253761) of NCBI accession number AECK01000005.1 [12].
(EPS)

S10 Fig Nineteen candidate genes not required for mating-type switching. Genes were selected based on their expression patterns in *O*. *polymorpha* or their known roles in other species. Deletion strains in a *MAT*α *ku80Δ* background were generated by electrotransformation. The *MAT* locus was PCR amplified from each strain before, and 24 h after, transfer from a YPD pre-induction culture into NaKG. The wildtype (WT) panels in this figure are reproduced from Fig 1C and Fig 6 because these assays were all done together.
(EPS)

S1 Table *O*. *polymorpha* genes induced and repressed on nitrogen-depleted (NaKG) vs. rich (YPD) media. (XLSX)

S2 Table Gene expression differences between *O*. *polymorpha MAT*a and *MAT*α haploid cells. (XLSX)

S3 Table Genes with highest expression differences between haploid (*MAT*a) and diploid (*MAT*a/α) *O*. *polymorpha* cells, in YPD media and NaKG media. (XLSX)

S4 Table O. *polymorpha* genes with largest expression differences in *efg1*Δ vs. WT cells in different media: (A) NaKG media plus ammonium sulfate (nitrogen-replete); (B) NaKG media (nitrogen-poor); (C) SD media (nitrogen-replete); (D) SD media without nitrogen (nitrogen-poor).
(XLSX)

S5 Table O. *polymorpha* genes with largest expression differences in (A) *pAOX-EFG1*, (B) *pAOX-STE12*, and (C) *pAOX-RME1*, vs. *pAOX* control upon induction of expression in methanol media.
(XLSX)

S6 Table Strains and plasmids used in this study. (XLSX)

S7 Table Primers used in this study. (XLSX)
