# Supplementary figures and images for: Flip/flop mating-type switching in the methylotrophic yeast Ogataea polymorpha is regulated by an Efg1-Rme1-Ste12 pathway
Source: PLoS Genet. 2017 Nov 27;13(11):e1007092. doi: 10.1371/journal.pgen.1007092 (PMC5720833; doi:10.1371/journal.pgen.1007092)

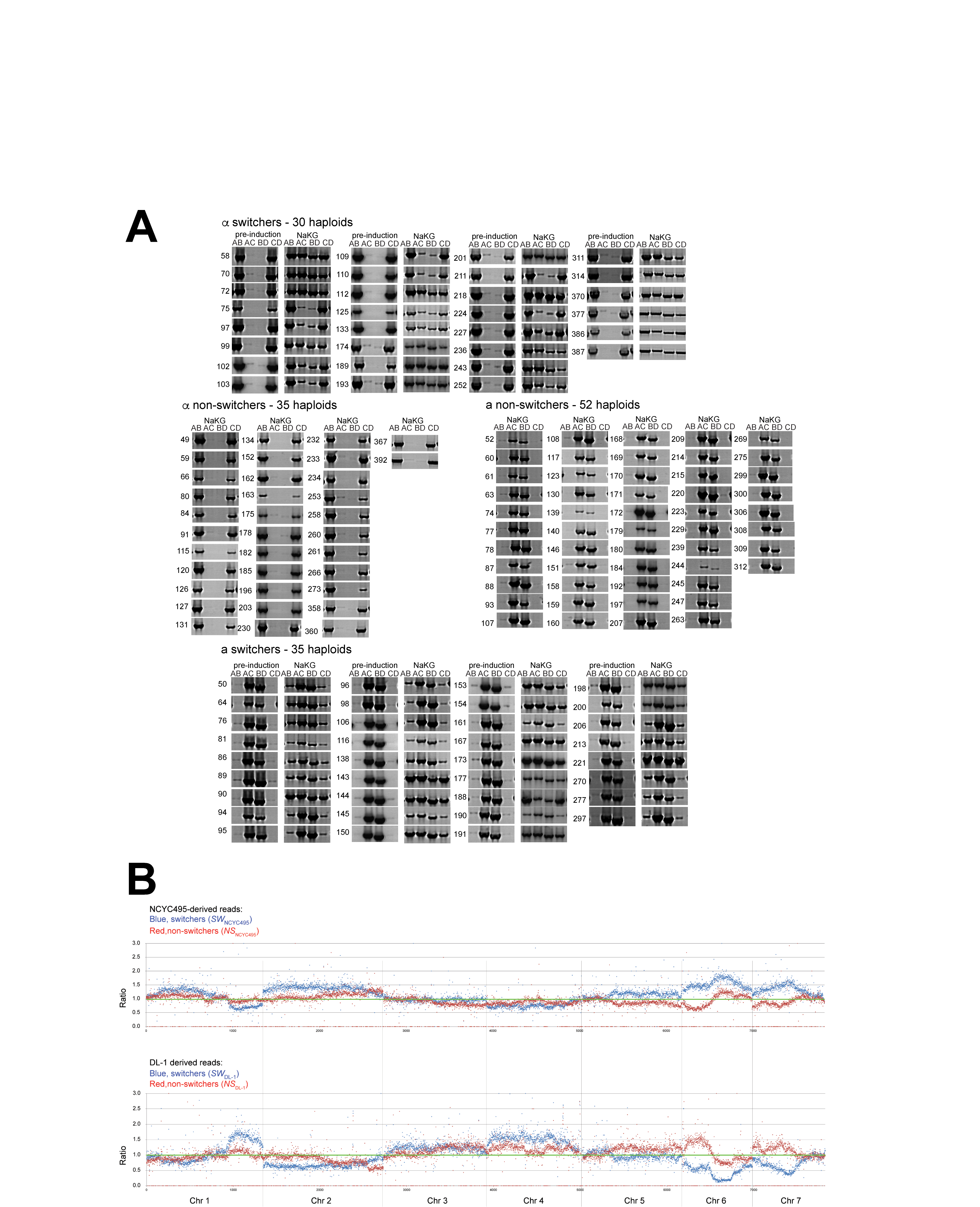

Supplement: S5 Fig — (A) Phenotypes of haploids used in bulk segregant analysis. Random spores isolated from a DL-1 x NCYC495 diploid were grown overnight in YPD before transfer to NaKG for 24 h. Gels show PCR analysis of the MAT locus to determine the original mating type and switching phenotype of the haploid isolates. Haploids were classified into four groups as shown. (B) Inheritance of genomic regions derived from NCYC495 and DL-1 in the sequenced pools of switching and non-switching progeny. The upper panel shows reads that mapped exclusively to the NCYC495 reference genome, and the lower panel shows reads that mapped exclusively to the DL-1 reference genome, from switcher (blue) and non-switcher (red) pools. The Y-axis is the ratio between the normalized number of mapped reads from a pool, relative to the number from the parental strain sample, in each of the 7824 genomic segments. Chromosome numbering and orientation follows the convention for O. polymorpha [12]. (TIF) [file pgen.1007092.s005.tif]
